# Measurement and Forecasting of High-Speed Rail Track Slab Deformation under Uncertain SHM Data Using Variational Heteroscedastic Gaussian Process

**DOI:** 10.3390/s19153311

**Published:** 2019-07-27

**Authors:** Qi-Ang Wang, Yi-Qing Ni

**Affiliations:** 1 State Key Laboratory for Geomechanics and Deep Underground Engineering & School of Mechanics and Civil Engineering, China University of Mining and Technology, Xuzhou 221008, China; 2 National Rail Transit Electrification and Automation Engineering Technology Research Center (Hong Kong Branch), Department of Civil and Environmental Engineering, The Hong Kong Polytechnic University, Hung Hom, Kowloon, Hong Kong, China

**Keywords:** uncertainty, measurement and forecasting, high-speed rail, structural health monitoring, fiber Bragg grating, Heteroscedastic Gaussian Process

## Abstract

Uncertainty in sensor data complicates the construction of baseline models for the measurement and forecasting (M&F) of high-speed rail (HSR) track slab deformation. Standard Gaussian process (GP) assumes a uniform noise throughout the input space. However, in the application to modelling of HSR structural health monitoring (SHM) data, this assumption can be unrealistic, because of its unique heteroscedastic uncertainty that is induced by dynamic train loading, electromagnetic interference, large temperature variation, and daily maintenance actions of railway track infrastructure. Therefore, this study firstly develops a novel online SHM system enabled by fiber Bragg grating (FBG) technology to eliminate electromagnetic interference on SHM data for continuous and long-term monitoring of track slab deformation, with the capacity of temperature self-compensation. To deal with different sources of uncertainty, the study explores Variational Heteroscedastic Gaussian Process (VHGP) approach while using variational Bayesian and Gaussian approximation for data modelling, estimation of the monitoring data uncertainty, and further data forecasting. The results demonstrate that the VHGP framework yields more robust regression results and the estimated confidence level can better depict the heteroscedastic variances of the noise in HSR data. Higher accuracy for both regression and forecasting is gained through VHGP and the position with maximum noise can be more accurately forecasted with a smooth varying confidence interval. Based on in-situ measurement data, the uncertainty levels for all sensors are estimated together with corresponding deformation profiles for the instrumented segment and three typical types of uncertainty are summarized during the M&F process of HSR track slab deformation.

## 1. Introduction

The rapid uptake of high-speed rail (HSR) has been largely due to its superior economic, social, and environmental benefits in comparison with other transport modes [1], and China’s HSR has witnessed significant development over the past 15 years. For the HSR ballastless track system, one type of low maintenance track [2,3] which was extensively used for HSR lines in China (e.g., Beijing-Shanghai HSR, Lanzhou-Xinjiang HSR, Beijing-Shenyang HSR, etc.), track slab infrastructure assets are crucial elements, and their sustained efficient and safe operation is essential for ensuring the running safety of high-speed trains. Structural health monitoring methods that can detect performance changes and precursors to failure are a critical tool for guaranteeing the efficient and safe operation. Generally, the main interests in the monitoring of the track slab are the whole-life-cycle deterioration [4,5,6] and the deformation under external excitation (e.g., natural disaster, dynamic train loading, etc.) or environmental effects during the operation of HSR lines [7,8,9]. This study focuses on the latter issue—deformation monitoring.

Maintaining the alignment of a rail track is essential for the smooth and safe passage of the rail vehicles, and ideal rail track guides the vehicles along a smooth path, following the design curvature. The deformation of track slab will inevitably have an influence on the track alignment and result in track irregularity since there is a rigid connection between the rail track and track slab, which can undermine the ride comfort and running safety of the vehicles. HSR track irregularity can be measured several times every month by specially designed rail track inspection vehicles and the versine (or mid-chord offset), where the lateral offset of the rail from the center of a string that is stretched between two ends touching the rail side is generally used to describe the track alignment or irregularity [10]. For a straight track, the versine should be zero, and the versine is a constant value for constant curves. However, these methods cannot provide real-time and long-term monitoring.

**Figure 1:**
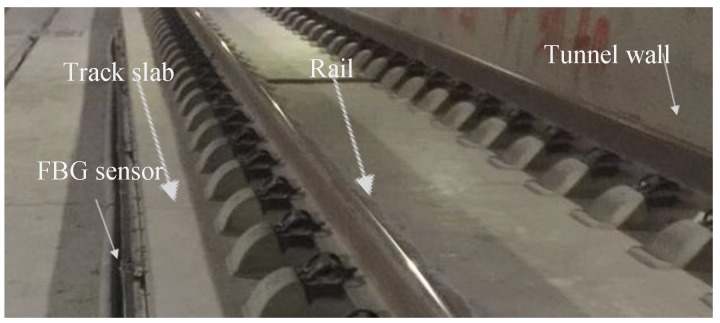
High-speed rail (HSR) slab track system in the tunnel.

The monitored segment of the ballastless track slab is located inside a large-section deep-buried railway tunnel of a high-speed railway (Figure 1). Cracks appear in the railway tunnel walls, and spalling of the concrete happens, especially in the arch vault and foot of the tunnel, due to the geological disaster of landslide induced by earthquakes. According to the measurement of CP III track control network, both lateral and vertical deformation occurs in the railway tunnel. The range of lateral displacement is +22 mm ~ +104 mm. The deformation of the whole tunnel would directly lead to the displacement of the track slab, which results in large lateral track irregularity. To restore the railway operation, the railway management departments immediately carried out the reconstruction of the ballastless track bed in the section with large deformation. It is necessary to deploy a real-time online deformation monitoring system for rail section in concern, so as to achieve real-time warning, when the track irregularity reaches a certain level in order to ensure the safe operation of high-speed railway. 

Although there have been many studies on deformation monitoring for civil infrastructure while using various techniques and devices, e.g., tape extensometers [11,12], total stations [13,14], photogrammetry [15,16], and three-dimensional (3D) laser scanners [17,18], these methods have their own disadvantages for HSR track slab deformation monitoring: the tape extensometer can only be used to measure the distance changes relative to a fixed point and must be manipulated manually; total station is vulnerable to environmental interferences; and, the laser scanning technique has low accuracy with an error level of around ±5 mm. Therefore, this study will firstly develop a novel online SHM system that is enabled by fiber Bragg grating (FBG) technology (immunity to electromagnetic interference in HSR) for continuous and long-term monitoring of track slab deformation. The devised FBG bending gauge sensor does not require an external power supply, and its sensing principle is converting a wavelength shift of FBG into a physical quantity to be measured, and thus will not be affected by electromagnetic interference. The traditional piezoelectric sensor converts the electrical signal into a physical quantity of interest, so it is subject to electromagnetic interference, and the phenomenon of frequency multiplication occurs in the frequency domain of the original measurement data. In addition, the technical advantages of the developed FBG sensor include the following: (i) high measurement accuracy: the strain accuracy can reach 1 με; (ii) suitable for harsh environment in HSR; (iii) suitable for remote sensing; (iv) small size (diameter of FBD is about 0.15 mm); and, (v) cheap price.

In terms of SHM of railways specifically, the data noise and uncertainty are mainly from (i) the dynamic train loading and the ground-borne vibration arising from the wheel/rail interface, train weight, change in supporting stiffness (e.g., regularly spaced sleepers), and irregularities in the wheel/rail geometry [19,20]. Besides, the vibration will be elevated if the running speed of the train is comparable with the natural Rayleigh wave speed in the supporting soil [21,22]; (ii) the HSR contact network of electric locomotives, with a voltage of 27,000, provides power for the train, which will inevitably generate electromagnetic interference to the piezoelectric sensors. The devised SHM in this study, which is based on FBG, in contrast, is immune to the electromagnetic interference. It will greatly reduce the data uncertainty and ensure the reliability of measurement data; (iii) the fiber Bragg grating wavelength of the FBG-based sensor is sensitive to the temperature change. Large temperature variation can create large uncertainty in the measurement data. The monitoring system is designed for years of continuous monitoring. The maximum temperature difference in summer can be 20 degrees, and the maximum temperature difference throughout the whole year can reach up to 50 degrees. Although the developed FBG sensor considers the temperature compensation, such a large temperature difference can still cause daily and seasonal fluctuation in measured deformations; and, (iv) other factors, such as demodulation error of demodulator and the influence of periodic inspection activities on the high-speed railway (maintenance personnel may kick on the sensor, which will cause data fluctuation). The aforementioned four types of uncertainties will result in heteroscedastic noise in HSR SHM data at different time periods. Therefore, developing a rational data modelling method to deal with such non-stationary SHM data with input-dependent noise is highly desired.

Gaussian process (GP) modelling [23,24,25] is a powerful statistical modelling framework that puts forward a structure for the covariance matrix of input variables to calculate a predictive distribution for output variable. The GP model is capable of capturing complex nonlinear relationships between input and output by employing Bayesian framework for covariance hyper-parameters training, and it can offer mean prediction for testing points with associated uncertainty levels (e.g., 95% confidence interval). A limitation of the standard (homoscedastic) GPs is the assumption of constant noise power throughout the input space, i.e., homoscedastic noise [26]. To release this assumption, Heteroscedastic Gaussian process (HGP), which was first proposed by Goldberg [27], on the other hand assumes that observation noise can vary and it aims to model the mean and variance distributions. In the framework of HGP, two-GP models have been proposed: one estimates the mean while the other captures the log-noise power. Making inferences for HGP is not as easy as standard GPs, because the predictive posterior distribution also accounts for the noise rates as the independent latent variables, which makes it intractable to analytically deal with the integral for the predictive posterior distribution. Therefore, a number of numerical methods have emerged, including Markov Chain Monte Carlo (MCMC), most likely HGP, sparse Gaussian process (referred to as GPz), and *maximum a posterior* (MAP) HGP. Goldberg et al. [27] proposed MCMC sampling to approximate the posterior distribution over the aforementioned two GPs. The solution converges to the exact posterior when the number of samples tends to infinity, but the computational cost is very high and it makes the model expensive to compute on large data sets. Kersting et al. [28] employed a most likely noise approach to approximate the posterior noise variance. The likelihood is maximized while using an iterative procedure that is similar to Expectation Maximization updates. However, the algorithm is not guaranteed to converge and it may instead oscillate. In most recent related works, Almosallam et al. [29] proposed a sparse Gaussian process (GPz) for heteroscedastc uncertainty estimation. In this framework, a Bayesian machine learning approach is employed to jointly optimize the model with respect to both the predictive mean and variance. The variance is an input-dependent function and it consists of two terms that capture different sources of uncertainty. The first term represents the intrinsic uncertainty about the mean function because of data density, while the second term is the uncertainty that is due to the intrinsic noise or the lack of precision/features in the training data. In order to achieving accurate predictions, GPz incorporates a sparsity-inducing prior to minimize the number of basis functions, to produce a sparse model representation. The MAP approach has been performed to maximize a penalized likelihood for the HGP approximation [30]. Le et al. [31] presented a non-parametric approach to estimate heteroscedastic noise by performing *maximum a posteriori* for regression with exponential families. However, MAPHGP provides a point estimate for noise, while the Variational Heteroscedastic Gaussian Process (VHGP) that is explored in this study is a fully Bayesian approach that variationally integrates out the GP for noise term. The VHGP firstly proposed by Lazaro-Gredilla and Titsias [32], is based on variational Bayes and the Gaussian approximation for accurate inference in HGP. The computational cost for HGP is greatly reduced and at the same time a high accuracy can be guaranteed. These features make VHGP particularly attractive for modelling and forecasting of SHM data corrupted with uncertainties, which can help to assess the amount and resolution of data required to reach desired uncertainty levels.

In this paper, a novel online SHM system is developed for monitoring the lateral deformation of track slabs by deploying an array of FBG bending gauges, which is able to continuously detect the deformation of track slabs and provide an automatic condition assessment of in-service railway. FBG bending gauges with temperature self-compensation are designed and fabricated, and the sensor array is verified in laboratory experiments before the implementation of the devised system in an in-service HSR line. This study explores a VHGP approach with variational Bayesian and Gaussian approximation for data modelling to handle the different sources of uncertainty in SHM data of track slabs, which quantifies the uncertainty level of the monitoring data and further makes forecasting on SHM data. Previous studies have proved that VHGP is superior to MAPHGP and MLHGP in various applications [32,33]. MCMC HGP typically needs several thousands of sample-generating runs before achieving stable results; thus, the computational cost is extremely high. HGP and MAP HGP both most likely provide a point estimate for noise in the iterative solution, while the Variational Heteroscedastic Gaussian Process (VHGP) gives rise to a full distribution result instead of a single point estimate. In this regard, the GPz method is quite similar to VHGP method, because it also generates full distribution results through a Bayesian machine learning approach. This paper will compare VHGP with the latest HGP model, i.e., GPz, to illustrate its advantages in dealing with SHM data for modelling and forecasting of the deformation of HSR track slabs. Laboratory and field tests will both be made to validate the proposed method.

The contributions of this study include:

(i) A novel online SHM system enabled by FBG bending gauges is developed for monitoring the lateral deformation of HSR track slabs, with the capacity of temperature self-compensation.
(ii) The study innovatively applies VHGP to deal with the SHM data for treatment of input-dependent noise, uncertainty qualification, and forecasting. The performance of two HGP models, namely VHGP and GPz, is compared for the first time.
(iii) A specific example of M&F for deformation of an in-service HSR track slab is provided. Uncertainty characteristics of SHM data for HSR specifically are discussed and the uncertainty levels of all in-situ sensors in the monitored segment are obtained, with a comprehensive analysis of uncertainty sources.

## 2. VHGP Framework

### 2.1. Standard GP Methodology

The GP is a supervised nonlinear regression algorithm lying within the class of Bayesian non-parametric models. Given a set of inputs $x={\left\{x_{n}\right\}}_{n=1}^{N}\in R^{N\times d}$ and a set of target outputs $y={\left\{y_{n}\right\}}_{n=1}^{N}$, where *N* represents the number of input or output. The underlying assumption is that *y* is generated by a function of the input *x*, plus additive noise $\varepsilon_{i}$

(1) $$y=f\left(x\right)+\varepsilon_{i}$$

where the independent noise term $\varepsilon_{i}$ is assumed a zero-mean and *σ*^2^-variance Gaussian prior. *y* has a Gaussian prior distribution with a zero-mean function (without loss of generality) and a positive-definite covariance function $k_{f}\left(x,x^{\prime };\theta_{f}\right)$ with the hyperparameters $\theta_{f}$. f = [*f*_1_, …, *f_N_*]*^T^* is defined as the evaluation of f(⋅) on the inputs x. Subsequently, a multivariate Gaussian prior has the form $p\left(f|X\right)=N\left(f|0,K_{N}\right)$, where ${\left[K_{N}\right]}_{ij}=k_{f}\left(x_{i},x_{j}\right)$. Thus, the Gaussian likelihood is $p\left(y|f\right)=N\left(y|f,\sigma^{2}I\right)$, where ***I*** is a unit matrix. While integrating out the latent function values, the marginal likelihood can be obtained as

(2) $$p\left(y|x,\theta \right)=N\left(y|0,K_{N}+\sigma^{2}I\right)$$

where θ represent hyperparameters. A maximum for the logarithm of marginal likelihood in Equation (2), also known as log-evidence, is sought to choose hyperparameters θ and $\sigma^{2}$. Finally, the predictive posterior distribution for a new sample $x_{*}$ is distributed normal with the following mean and variance $y_{*}\sim N\left(\mu_{*},\sigma_{*}^{2}\right)$ ($y_{*}$ represents the target output for a new sample $x_{*}$; $\mu_{*}$ is the mean value of $y_{*}$, and $\sigma_{*}^{2}$ is the variance of $y_{*}$):

(3) $$\mu_{*}=k_{*N}{\left(K_{N}+\sigma^{2}I\right)}^{-1}y$$

(4) $$\sigma 2_{*}=k_{**}-k_{*N}{\left(K_{N}+\sigma^{2}I\right)}^{-1}k_{*N}+\sigma^{2}$$

where ${\left[k_{*N}\right]}_{j}=k_{f}\left(x_{*},x_{j};\theta_{f}\right)$, $y={\left[y_{1},y_{2},\ldots ,y_{n}\right]}^{T}$ and $k_{**}=k_{f}\left(x_{*},x_{*};\theta_{f}\right)$.

### 2.2. Heteroscedastic GP Model

To define the heteroscedastic GP (HGP) model, a Gaussian prior is placed on the noise term $\varepsilon_{i}$:$\varepsilon_{i}\sim N\left(0,r\left(x_{i}\right)\right)$, in which the variance *r*(*x*) of the noise can vary at each input *x*. $r\left(x\right)=e^{g\left(x\right)}$ is parametrized to ensure positivity and a GP prior is placed as $g\left(x\right)=GP\left(\mu_{0},k_{g}\left(x,x^{\prime };\theta_{g}\right)\right)$.

Once the parametric forms of $k_{f}\left(x,x^{\prime }\right)$ and $k_{g}\left(x,x^{\prime }\right)$ are determined, the HGP model will be specified and it only depends on $\mu_{0}$ and the covariance hyperparameters: $\theta_{f}$ and $\theta_{g}$. For VHGP, since $\mu_{0}$ is explicitly considered, the scale of the noise term can be controlled. 

### 2.3. MV Bound for HGP and Optimization

VHGP is more flexible when compared with the standard GP. However, it is analytically intractable. Thus, variational approximation is proposed to deal with the computational difficulty. While it is impossible to calculate the marginal log-likelihood analytically for HGP, it can be lower bounded by variational approximation.

The definition of standard variational approximation follows:

(5) F(q(f),q(g))=log(p(y))−KL(q(f)q(g)‖p(f,g|y))

Since Kullback–Leibler (KL) divergence [34,35,36] is non-negative, it is obvious that the evidence log(p(y)) is lower bounded by *F*, i.e., F(q(f),q(g))≤logp(y) for any variational distributions q(f) and q(g). The objective is to maximize the bound *F* with respect to q(f) and q(g), and it is equivalent to minimize the KL divergence with the fact that the evidence has no dependence on q(f) and q(g), which is to find the best approximation to the posterior distribution, according to the definition of the KL divergence. 

*F* depends on both q(f) and q(g). For simplification, the Marginalized Variational (MV) bound will be obtained by marginalizing out q(f) to remove its dependence. The optimal distribution $q^{*}\left(f\right)$ can be obtained through the variational Bayesian theory to maximize F(q(f),q(g))

(6) $$\underset{q\left(f\right)}{q^{*}\left(f\right)=argmaxF}=\frac{p\left(f\right)}{Z\left(q\left(g\right)\right)}e^{\int q\left(g\right)\log p(y|f,g)dg}$$

where Z(q(g)) is a constant, i.e., $Z\left(q\left(g\right)\right)=\int e^{\int q\left(g\right)\log p\left(y|f,g\right)dg}p\left(f\right)df$. The MV bound can be obtained by inserting $q^{*}\left(f\right)$ back to F(q(f),q(g)):

(7) F(q(g))=logZ(q(g))−KL(q(g)‖p(g))

which removes the dependence on q(f).

If q(g) is restricted to be a multivariate normal distribution, i.e., N(g|μ,Σ), the MV bound for HGP model can be rewritten as

(8) $$\begin{array}{l} F\left(\mu ,\Sigma \right)=\log\int e^{\int N\left(g|\mu ,\Sigma \right)\log p\left(y|f,g\right)dg}N\left(f|0,K_{f}\right)df\\ -\mathrm{KL}\left(N\left(g|\mu ,\Sigma \right)\|N\left(g|\mu_{0}1,K_{g}\right)\right) \end{array}$$

where ***K****_f_* and ***K****_g_* represent the covariance matrices of *f* and *g*. After the simplification, the MV bound for VHGP can be obtained, as follows:

(9) $$\begin{array}{l} F\left(\mu ,\Sigma \right)=\log N\left(y|0,K_{f}+R\right)-\frac{1}{4}tr\left(\Sigma \right)\\ -\mathrm{KL}\left(N\left(g|\mu ,\Sigma \right)\|N\left(g|\mu_{0}1,K_{g}\right)\right) \end{array}$$

where ***R*** is a diagonal matrix with elements ${\left[R\right]}_{ii}=e^{[\mu {]}_{i}-[\Sigma {]}_{ii}/2}$.

The MV bound depends on *N+N(N+1)/2* free variational parameters (defining μ and Σ). According to Gaussian approximation theory, the stationary equations $\frac{\partial F\left(\mu ,\Sigma \right)}{\partial \mu }=0$ and $\frac{\partial F\left(\mu ,\Sigma \right)}{\partial \Sigma }=0$ must be satisfied at any local or global maximum, and the two equations are obtained as follows after manipulation:

(10) $$\mu =K_{g}\left(\Lambda -\frac{1}{2}I\right)1+\mu_{0}1,\;\Sigma^{-1}={K_{g}}^{-1}+\Lambda$$

for some semi-positive definite matrix Λ. Thus, both μ and Σ depend on Λ, and only *N* diagonal elements are needed to define Λ. Therefore, after reparametrization, the number of free variational parameters for optimization is reduced to be *N*. Eventually, the MV bound F(μ(Λ),Σ(Λ))=F(Λ) needs to be maximized with respect to the *N* variational parameters in Λ. This is advantageous, both from a computational point of view and an optimization point of view. At the same time, *F* can be maximized with respect to the hyperparameters θ through Type-II Maximum Likelihood. The whole optimization is non-linear and gradient-based procedures, and the derivatives with respect to (Λ,θ) can be analytically computed.

### 2.4. Predictive Posterior Distribution for VHGP

Given training data, $p(y_{*}|x_{*},D)$ represents the predictive distribution for a new test output $y_{*}$. $q^{*}\left(f\right)q\left(g\right)$ is viewed as a good approximation to the posterior distribution p(f,g|D), the mean and variance of *p*(y_*_|x_*_,D) can be analytically calculated.

$q^{*}\left(f\right)$ can be calculated according to Equation (6):

(11) $$q^{*}\left(f\right)=N(f|K_{f}\alpha ,K_{f}-K_{f}\left(K_{f}+R{)}^{-1}K_{f}\right)$$

where $\alpha ={\left(K_{f}+R\right)}^{-1}y$. Through the variational approximation, the posterior distribution for $f_{*}=f\left(x_{*}\right)$ is

(12) $$q\left(f_{*}\right)=\int^{\text{​}}p\left(f_{*}|x_{*},x,f\right)q^{*}\left(f\right)df$$

where $a_{*}=k_{f_{*}}^{T}\alpha$ and $c_{*}^{2}=k_{f_{**}}-k_{f_{*}}^{T}{\left(K_{f}+R\right)}^{-1}k_{f_{*}}$. Similarly, the posterior distribution of $g_{*}=g\left(x_{*}\right)$ is obtained by:

(13) $$q\left(g_{*}\right)=\int^{\text{​}}p\left(g_{*}|x_{*},x,g\right)q\left(g\right)dg=N\left(g_{*}|\mu_{*},\sigma_{*}^{2}\right)$$

where $\mu_{*}=k_{g_{*}}^{T}\left(\Lambda -\frac{1}{2}I\right)1+\mu_{0}$ and $\sigma_{*}^{2}=k_{g_{**}}+k_{g_{*}}^{T}{\left(K_{g}-\Lambda^{-1}\right)}^{-1}k_{g_{*}}$. Consequently, the distribution for $y_{*}$ is estimated by:

(14) $$\begin{array}{l} q\left(y_{*}\right)=\int^{\text{​}}\int^{\text{​}}p\left(y_{*}|g_{*},f_{*}\right)q\left(f_{*}\right)q\left(g_{*}\right)df_{*}dg_{*}\\ =\int^{\text{​}}N\left(y_{*}|a_{*},c_{*}^{2}+e^{g_{*}}\right)N\left(g_{*}|\mu_{*},\sigma_{*}^{2}\right)dg_{*} \end{array}$$

Although the above expression is not analytically tractable, the mean and variance of the posterior distribution can be analytically calculated: $E_{q}\left[y_{*}|x_{*},D\right]=a_{*}$ and $V_{q}\left[y_{*}|x_{*},D\right]=c_{*}^{2}+e^{\mu_{*}+\sigma_{*}^{2}/2}$. 

## 3. SHM System

### 3.1. Development of FBG Bending Gauge

**Figure 2:**
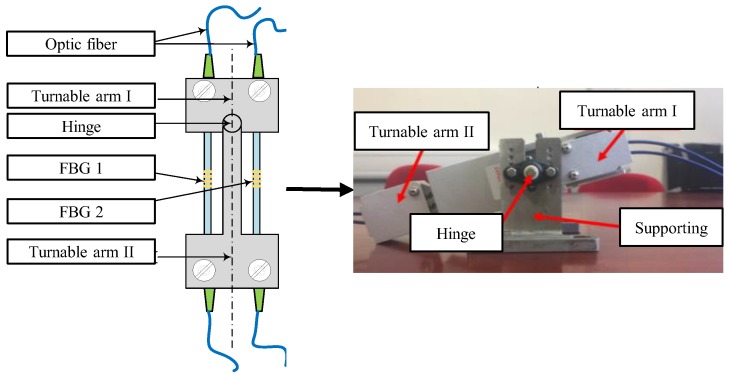
FBG bending gauge sensor.

Figure 2 shows the design of an FBG bending gauge [37], and it consists of two turnable arms and a revolution joint with one rotational degree of freedom, which allows for the two arms to rotate at an angel within ±1°. Two pre-tensioned FBGs are mounted on opposite sides in regard to the centerline of the turnable arm, which allows for the measurement of angle change. The sensor has a total length of 25 cm, a width of 5 cm, and a thickness of 5 cm. The length of the two FBGs is the same and both are 12 cm. The length of optical fiber grid is 1 cm, which determines the accuracy of strain demodulation. When there is an angle of rotation, the sensor will experience either tensile or compressive strains, which will induce wavelength changes of two FBGs. Finally, displacement and 6m versine values can be derived, according to the angle information (described in Section 3.2).

**Figure 3:**
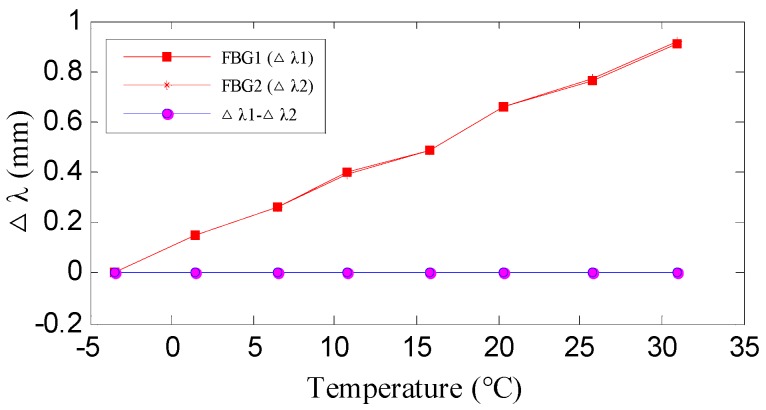
Temperature self-compensation of the FBG bending gauge.

The reason for the design with two FBGs is to eliminate the influence of temperature on FBG wavelength shift. When a rotation occurs, the FBG on one side undergoes a tensile force while the FBG on the other side undergoes a compressive force. The wavelength shifts are induced by both strain and temperature variances. Since the two FBGs are spatially close to each other, the ambient temperature variation that is experienced by them can be assumed to be identical. Thus, by subtracting the two FBG’s wavelength, the temperature induced wavelength shift will be eliminated, and the measurement subtraction is fully resulting from strains. An experiment was carried out to verify the temperature self-compensation capacity of the devised FBG bending gauge. The sensor was placed in a temperature chamber to wavelength shift under different temperature. The results (Figure 3) show that the wavelength shifts that were measured from the two FBGs are almost identical, and their subtraction can be plotted as a nearly straight zero-value line, which is not affected by temperature change. It demonstrates that the FBG bending gauge has excellent performance in compensating the effect due to temperature change.

### 3.2. SHM System for Track Slab Deformation Monitoring

In the longitudinal view, the FBG bending gauges are implemented at nearly equal intervals along the longitudinal direction of the track slabs. These FBG bending gauges are interconnected by rigid rods to form a chain that stretches in alignment with the track slabs and deforms in the same manner as the track slabs do.

**Figure 4:**
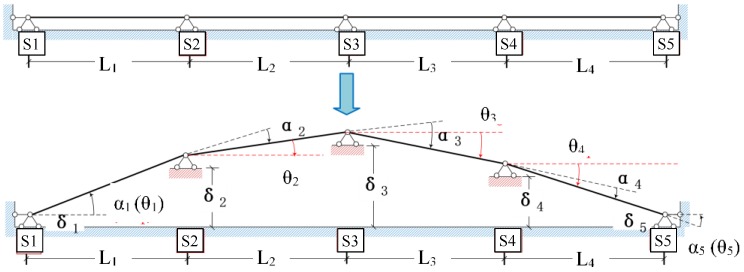
Displacement estimation based on measured rotation angles.

When deformation occurs in the track slab, the displacement of each FBG bending gauge can be calculated from the angles of rotation relative to its two adjacent bending gauges, as shown in Figure 4. Eventually, a configuration of the chain after deformation can be derived with the aid of an appropriate algorithm to indicate the displacement profile of the track slabs.

Consider a chain that formed with FBG bending gauges at distances denoted as {*L*_1_, *L*_2_, …, *L_n_*_−1_}. We can derive the absolute rotation angles {*θ*_1_, *θ*_2_, …, *θ_n_*} and the displacements {*δ*_1_, *δ*_2_, …, *δ_n_*} at the measuring points by using the measured relative rotation angles {*α*_1_, *α*_2_, …, *α_n_*}. From the geometric relationship, the following equations can be written for the *i*th bending gauge:

(15) $$L_{i}\tan\theta_{i}=\delta_{i+1}-\delta_{i}$$

(16) $$\alpha_{i}=\theta_{i}-\theta_{i-1}$$

It is assumed here that the rotation angle is positive if it is counterclockwise and negative if it is clockwise for both $\alpha_{i}$ and $\theta_{i}$ since the rotation angles would not be practically too large. Equation (15) is valid for *i =* 1, …, *n* − 1, while Equation (16) is valid for *i =* 2, …, *n* − 1. With $\alpha_{1}=\theta_{1}$, Equations (15) and (16) can be rewritten as follows for all the measuring points in matrix form:

(17) $${\left[\begin{array}{ccccccc} -1 & 1 & 0 & & 0 & 0 & 0\\ 0 & -1 & 1 & \cdots & 0 & 0 & 0\\ 0 & 0 & -1 & & 0 & 0 & 0\\ & \vdots & & \ddots & & \vdots & \\ 0 & 0 & 0 & & -1 & 1 & 0\\ 0 & 0 & 0 & \cdots & 0 & -1 & 1 \end{array}\right]}_{\left(n-1\right)\times n}\left[\begin{array}{c} \delta_{1}\\ \delta_{2}\\ \delta_{3}\\ \delta_{n-1}\\ \delta_{n} \end{array}\right]=\left[\begin{array}{c} L_{1}\tan\theta_{1}\\ L_{2}\tan\theta_{2}\\ L_{3}\tan\theta_{3}\\ L_{n-2}\tan\theta_{n-2}\\ L_{n-1}\tan\theta_{n-1} \end{array}\right]$$

(18) $${\left[\begin{array}{ccccccc} 1 & 0 & 0 & & 0 & 0 & 0\\ -1 & 1 & 0 & \cdots & 0 & 0 & 0\\ 0 & -1 & 1 & & 0 & 0 & 0\\ & \vdots & & \ddots & & \vdots & \\ 0 & 0 & 0 & & 1 & 0 & 0\\ 0 & 0 & 0 & \cdots & -1 & 1 & 0\\ 0 & 0 & 0 & & 0 & -1 & 1 \end{array}\right]}_{n\times n}\left[\begin{array}{c} \theta_{1}\\ \theta_{2}\\ \theta_{3}\\ \theta_{n-1}\\ \theta_{n} \end{array}\right]=\left[\begin{array}{c} \alpha_{1}\\ \alpha_{2}\\ \alpha_{3}\\ \alpha_{n-1}\\ \alpha_{n} \end{array}\right]$$

**Figure 5:**
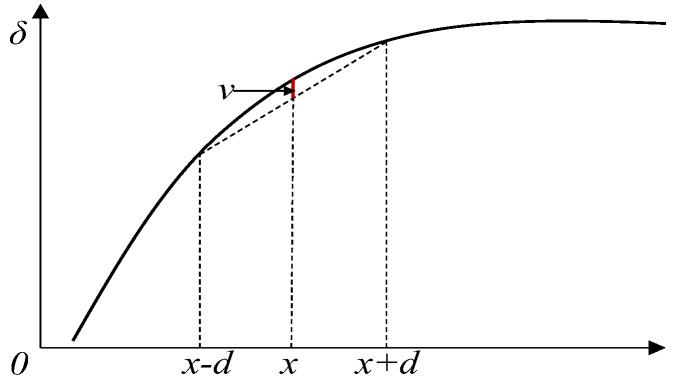
Relationship between versine and displacement profile.

The rank of the coefficient matrix in Equation (17) is *n* − 1, which is insufficient to obtain a deterministic solution. Therefore, the first FBG bending gauge is fixed with $\delta_{1}=0$. As for Equation (18), it can be easily seen that the coefficient matrix is full-rank, which indicates that there is a deterministic solution. In this way, the displacement profile can be derived from the measured relative rotation angles. As shown in Figure 5, the relationship between versine and displacement profile, where *d* is the spacing between two sensors, and the solid line represents the displacement profile. Thus, according to Equation (19), the *2d-*chord versine can be calculated.

(19) $$v\left(x\right)=\delta \left(x\right)-\frac{\delta \left(x-d\right)+\delta \left(x+d\right)}{2}$$

The sensors in this study are mounted on the side of the track slab, spaced at 3 m, and thus 6 m-chord versine will be adopted. Different limit state thresholds of the versine are required in different performance evaluation, according to the maintenance regulations for ballastless track lines of high-speed rail in China. When the value of 10 m-chord versine is greater than 8mm, temporary repair shall be carried out for the track. In this study, warning is expected to be alarmed upon the exceedance of the measured versine over a certain threshold.

After the SHM system is installed on a target section of track slabs, the real-time monitoring data will be uploaded onto a cloud database for storage through the cellular network. A front-end website has been developed while using Python and Matlab for real-time data display and visualization. The versine deformation that was measured from all FBG bending gauges will be updated after a period of time and it can be viewed directly online.

## 4. Case Study

### 4.1. Laboratory Test Data

**Figure 6:**
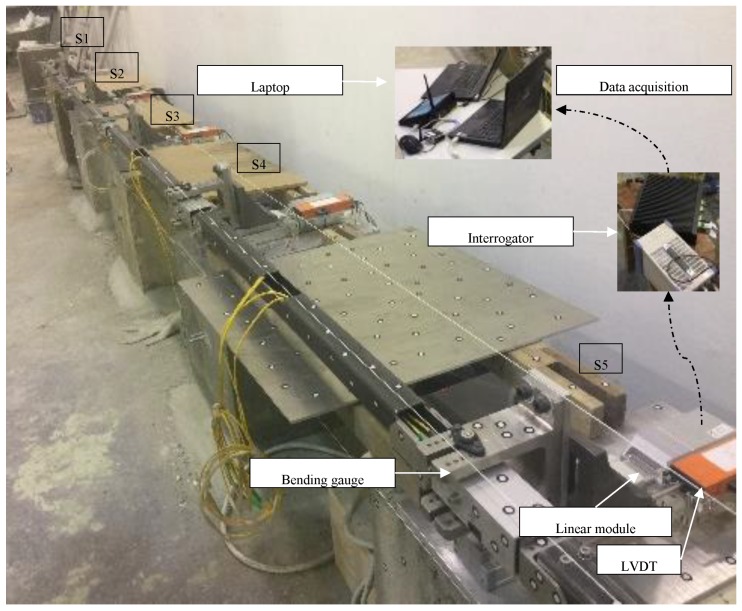
Laboratory test platform.

A test platform is constructed in the laboratory to verify the capability and feasibility of the SHM system enabled by FBG bending gauges (Figure 6). Five concrete blocks (0.5 m × 0.5 m × 0.5 m) are fixed on the ground to represent track slabs. The platform is built to simulate different deformation patterns of track slabs in railway tunnels: An electronic linear module is attached at each concrete block for generating transverse movements in a range of ±25 mm to simulate different deformation patterns of track slabs. The electronic linear modules receive commands from the control panel through a ZigBee wireless node to actuate displacement. Five FBG bending gauges (S1, S2, S3, S4, and S5) are connected by 3 m long rigid rods and then mounted on the concrete blocks to monitor the transverse deformation. The sampling rate is 1 datum per minute, which is sufficient for the track slab deformation monitoring induced by landslide. Verification experiments using LVDTs (with a measurement range of ±25 mm) as reference sensors have been carried out, which demonstrate that the SHM system enabled by FBG bending gauges satisfies the required accuracy of measuring different patterns of track slab deformation. An FBG interrogator, connected with 5 FBG bending gauges, is controlled by a laptop to collect and store the FBG wavelength shift. With the acquired wavelength shift, the displacement and versine will be derived while using the equations that are given in the previous section.

**Figure 7:**
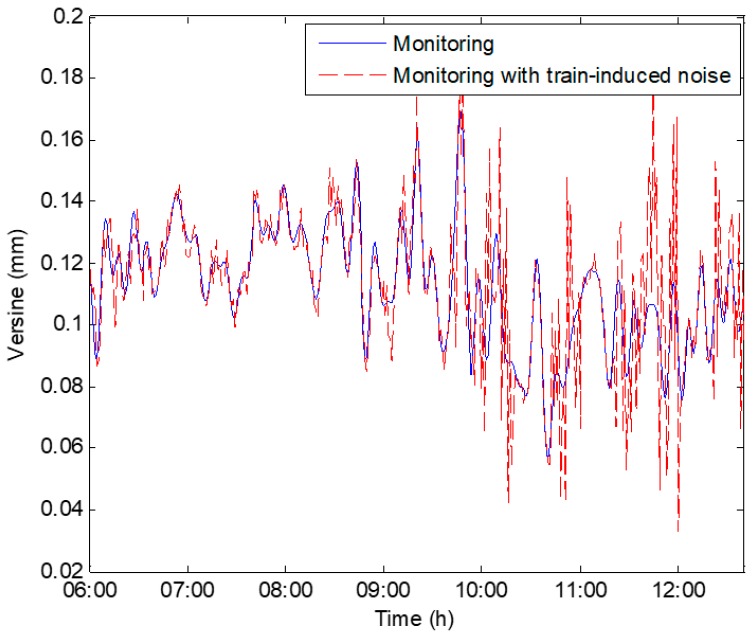
Data with the train-induced noise for VHGP training.

The laboratory data represent the real deformation data of the simulated track. However, for the in-situ monitoring, there exists the influence of passing trains, which will further introduce non-stationary noise to the data. Artificial random white noise with varying signal to noise ratio (SNR) is added to the data in the laboratory in accordance with the passing train time schedule in order to simulate the effect of passing trains on the experimental data volatility. Data from S3 on 17 August 2017 are given for illustration in Figure 7. Before 10:00 am, the passing train is less with a low noise level. More trains will pass after 10:00 am, and thus the SNR is increased accordingly. The data with train-induced noise will be used for the training data set, and the validity and feasibility of the VHGP method will be verified by comparison with the measurement data without train noise.

#### 4.1.1. VHGP Regression Performance

**Figure 8:**
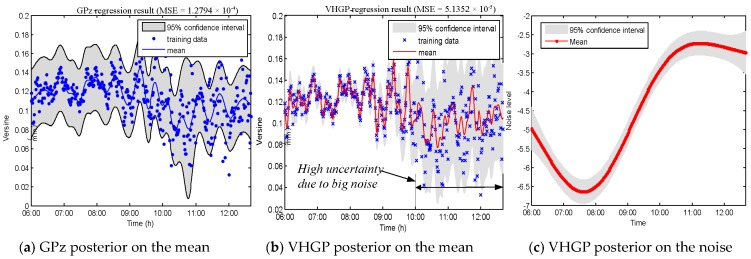
GPz and Variational Heteroscedastic Gaussian Process (VHGP) regression results on laboratory data: (**a**) mean distribution by GPz; (**b**) mean distribution by VHGP; and, (**c**) log-noise distribution by VHGP.

Figure 8 shows how the VHGP performs on the laboratory data, where real deformations are known (data from S3 on 17 August 2017). Automatic Relevance Determination Squared Exponential (ARD SE) covariance function is employed for *f*(*x*) and *g*(*x*). It is shown that the mean squared error (MSE) of the VHGP ($5.1352\times 10^{-5}$) is significantly smaller than that of GPz ($1.2794\times 10^{-4}$). VHGP and GPz regression works are carried out for other sensors, i.e., S2 and S4 in order to prove that this conclusion holds for other sensors in the laboratory test (since S1 and S5 are the reference sensors, the corresponding data are not analyzed). Table 1 shows a MSE comparison between GPz and VHGP for all the laboratory sensors. According to this table, it can be concluded that the regression errors of VHGP are much smaller than those of the GPz method for all sensors. The VHGP has higher regression accuracy. In addition, as shown in Figure 7, the noise in the second half of the data segment (after 10:00 am) is relatively bigger. The estimated confidence interval by VHGP gets wider, as shown in Figure 8b. The corresponding estimated noise level (VHGP) also increases, as shown in Figure 8c. However, the confidence interval of GPz evaluation does not change much, as seen in Figure 8a. Therefore, VHGP can better capture the heteroscedastic variances of the noise for the laboratory data, and the estimated confidence interval varies accordingly.

#### 4.1.2. VHGP Forecasting Performance

**Figure 9:**
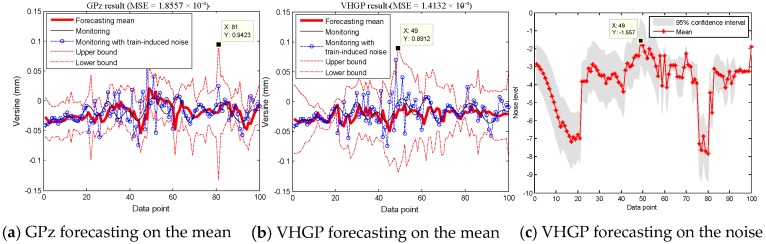
GPz and VHGP forecasting results on laboratory data: (**a**) mean distribution by GPz; (**b**) mean distribution by VHGP; and, (**c**) log-noise distribution by VHGP.

High accuracy can be gained when there are actual data to modify the deviation during the regression process. There is no guarantee that a better forecast result can be obtained for VHGP. Therefore, the verification test is then conducted to compare the prediction performance of the two methods. The one-step ahead forecast is carried out by using two methods in the same way: 300 data points are used as the training set to predict the measurement value for the next data point. The one-step ahead forecasting will be repeatedly made for 100 times to obtain the predictions for the next 100 data points. The forecasting mean with its 95% confidence interval and posterior distribution on the noise for S2 are shown in Figure 9 for illustration.

It demonstrates that both of the models capture the time-varying nature of the measurement noise, and the confidence interval width varies throughout the input space. The MSE of VHGP ($1.4132\times 10^{-4}$) is slightly smaller than that of GPz ($1.8557\times 10^{-5}$). However, there is some fundamental difference in the forecasting curves between VHGP and GPz that can only be revealed when taking a closer look at the training data and the forecasting results. As for the maximum noise, the GPz’s forecasting location is at data point 81 marked in Figure 9a. The maximum noise location estimated by VHGP is at data point 49 marked in Figure 9b. By comparing the measuring data before and after adding white noise, it can be found that real position for the maximum noise is near data point 49. Therefore, the VHGP framework yields more robust forecasting with more accurate maximum noise position being indicated in comparison with the GPz modelling approach.

### 4.2. Field Data

**Figure 10:**
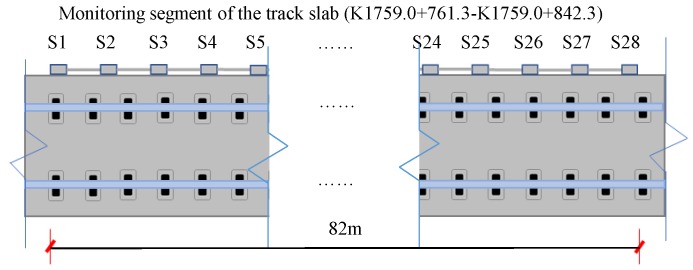
Sensor layout for the monitoring track slabs.

**Figure 11:**
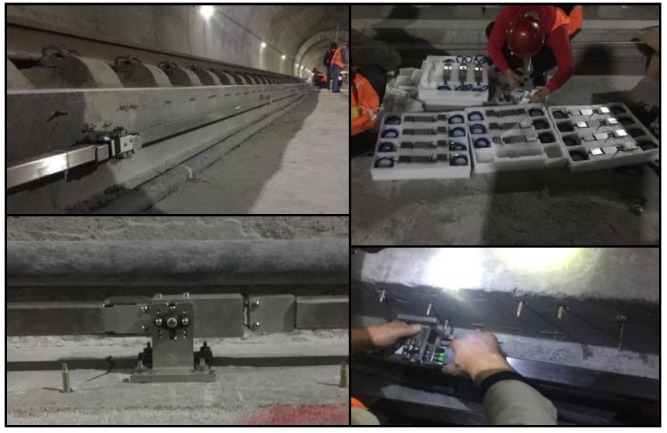
Implementation of SHM system.

After validating VHGP as a satisfactory approximation to the exact posterior for M&F of the laboratory test, in this section its regression and forecasting ability on the field monitoring data will be examined. The devised SHM system has been implemented on a segment of in-service track slabs inside a HSR tunnel. A total of 28 bending gauges were installed on one side of the track slabs to cover a length of 81 m in the longitudinal direction, with rigid rods of 3m long each connecting two adjacent FBG bending gauges (Figure 10 and Figure 11).

The real-time monitoring data are uploaded onto a cloud database for storage through the cellular network. The sampling rate is 1 datum per minute for all 28 bending gauges, which is sufficient for the track slab deformation monitoring, since the landslide induced deformation is a slow-varying process. Additionally, the low sampling rate is suitable, since the cellular network in a railway tunnel is usually inadequate for broadband data transmission. A front-end website has been developed using Python and Matlab for real-time data display and visualization. The versine deformations measured from all 28 bending gauges are updated every minute and they can be viewed directly online.

#### 4.2.1. Regression and Forecasting Performance

**Figure 12:**
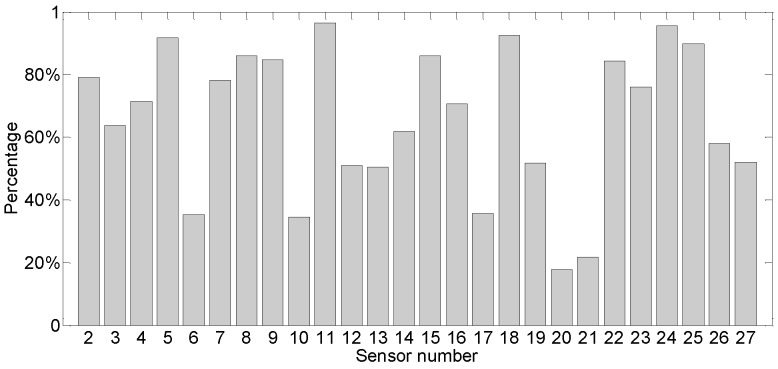
Accuracy improvement by VHGP as compared with GPz.

The study focuses on the long-term trend of deformation since the deformation in the instrumented segment of the track slabs (induced by slow landslide) is a slow-varying process. Therefore, 400 h of data are chosen for analysis (from 4 January 2018). First of all, the data preprocessing of the moving average is carried out to reduce the cost of calculation, and the average of every 60 data points (one-hour data) is taken. Finally, 400 data points are obtained for VHGP regression. The comparison results on the regression between VHGP and GPz for all sensors are given in Figure 12 (S1 and S28 are used as reference sensors), according to the mean squared error (MSE) of the model. The percentage of accuracy improvement is estimated through Equation (12). Basically, the MSE of VHGP is smaller than that of the GPz model for all sensor units and higher accuracy for regression is gained by using VHGP. For different sensors, the accuracy improvement level varies, with an average increase of 66.1%. Among them, the accuracy of S11 increases the most, up to 96.5% percent, and that of S20 increases the least, by about 17.8%.

(20) $$P=\frac{MSE_{GPz}-MSE_{VHGP}}{MSE_{GPz}}$$

**Figure 13:**
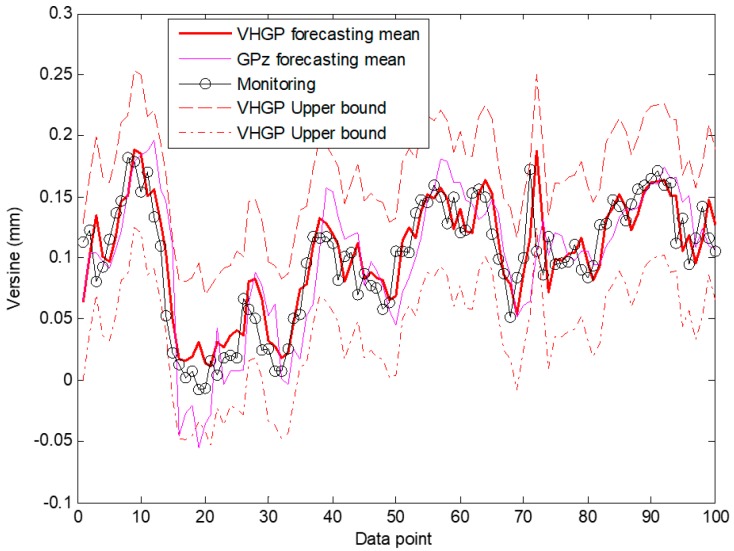
GPz and VHGP forecasting results for S18.

In addition, the measurement history data can be used to forecast the deformation in the next few days. If the predicted deformation value is larger than a certain threshold, early warning will be alarmed and preventive measures can be taken in advance. Similar to the laboratory data forecasting processing, one-step ahead forecasting is done, with 300 data points as the training set. According to the estimation results, VHGP is slightly superior to GPz in general, with a forecasting accuracy increase of 21.24% on average for all sensors. The improvement is not as high as that of regression, and it is most likely that the sparse algorithm of GPz improves the generalization ability of this model for forecasting. Among all sensors, the accuracy improvement rate of S8 is the largest, with the percentage of 56.65%. MSEs for VHGP and GPz are $5.1062\times 10^{-4}$ and $1.1799\times 10^{-3}$, respectively. Figure 13 shows the forecasting results by the two methods. It can be found that the forecasting mean of VHGP is more consistent with the real measurement values, especially at the peaks of the curve.

#### 4.2.2. Uncertainty Analysis

**Figure 14:**
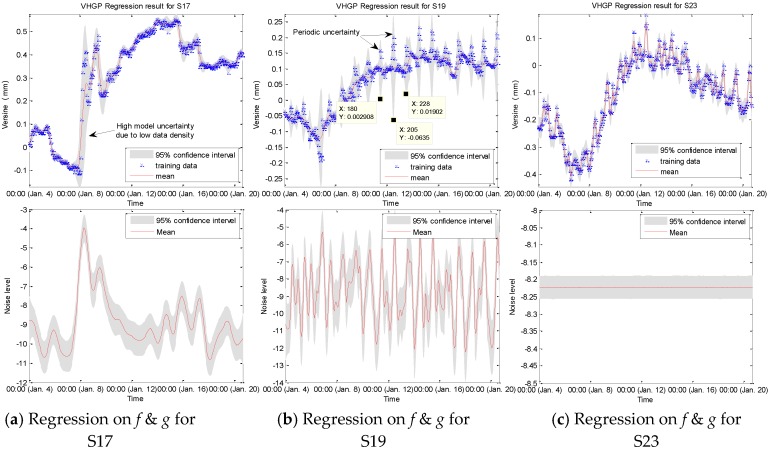
VHGP regression results on field data: (**a**) mean and log-noise distribution for S17; (**b**) mean and log-noise distribution for S19; and, (**c**) mean and log-noise distribution for S23.

The anti-interference capability of each sensor is different and the uncertainty in each sensor dataset varies due to the difference in sensor manufacturing error and field interference. Figure 14 shows three typical types of uncertainty.

For the sensor S17, as shown in Figure 14a, the uncertainty of model is relatively large from January 8 to January 10, which is caused by low data density. During this period, monitoring data changed considerably. As a result, the slope of the data curve sharply increases, resulting in this low data density phenomenon. There are a total of 48 data points from January 8 to January 10 for S17. However, during this period, the slope of the data fitting is relatively large on the monitoring data, resulting in a large distribution space of these 48 data, which ranges from −0.1 mm to 0.5 mm. Therefore, the data density in this space is reduced accordingly. Through comparison, trends in other sensor data are consistent with S17 at the same time period, but the slope is not as big as S17. When considering this consistence, it can be judged that there is a certain deformation in the monitoring segment during this period.

For the sensor S19, as shown in Figure 14b, there is periodic uncertainty in the model (between 11:00 am and 12:00 pm per day). It is found that the period is about 24 h, which is exactly one day. This phenomenon can also be found in other sensors (e.g., S10 and S11, etc.). It is mainly caused by the passing of trains on a regular basis. Between 11.00 am and 12:00 pm per day, it is the rush hour of the passing trains, which causes the data to greatly and periodically fluctuate.

For other sensors, such as S23 shown in Figure 14c, they have good stability with good anti-interference performance. The width of the confidence interval changes very little throughout the input space, and the noise level is basically unchanged, and the uncertainty level is stable, which shows a good robustness of the monitoring data.

**Figure 15:**
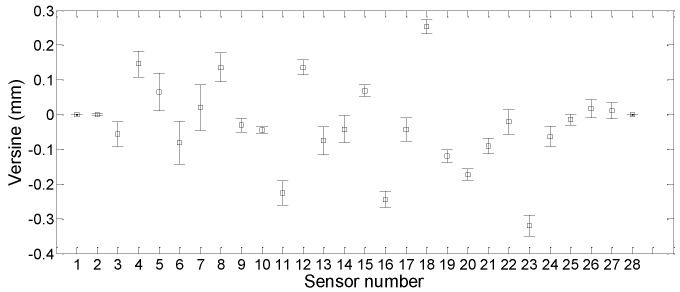
Model uncertainty levels for all sensors.

**Figure 16:**
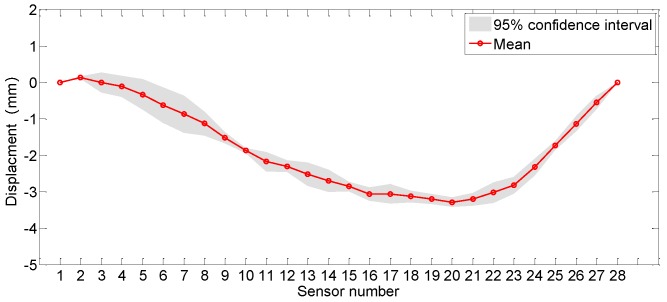
Deformation profile for the instrumented segment.

The purpose of data regression in this study is to quantify the uncertainty level of each sensor, to figure out the sensors with the maximum data volatility and the sensors with the most stable performance. Figure 15 shows the average values of the uncertainty levels of all the sensors on 6 January 2018, with the associated displacement profile being given in Figure 16. In Figure 16, the evaluated mean and 95% confidence interval of the displacement for the instrumented segment of the track slabs are given. It shows that the maximum displacement occurred at the sensor S3, with the value of 3.2898 mm.

In Figure 15, the length of error bar represents the magnitude of the variance, and the vertical coordinate at the midpoint represents the mean versine value of the sensor’s measurement. It demonstrates that the sensors with high data volatility are S5, S6, and S7. The most stable ones are S2, S10, and S15. During the modelling and forecasting for deformation of in-service HSR track slabs, the data of sensors with good stability shall be fully considered to obtain more accurate measurement results.

## 5. Conclusions

A novel online SHM system using FBG sensing technology has been developed, tested, and implemented for HSR track slab deformation monitoring. FBG bending gauges with temperature self-compensation capacity have been devised specifically for the deformation monitoring task and they are validated by laboratory test before the implementation of this system in an in-service HSR line. The SHM system can greatly eliminate the data uncertainty caused by electromagnetic interference in HSR applications since FBG is immune to electromagnetic interference.

To properly deal with the different sources of uncertainty, the study innovatively applies VHGP for SHM data modelling, which enjoys full Bayesian, non-parametric, probabilistic modelling, principled learning of free variational parameters with dimension reduction, and heteroscedasticity. The uncertainty level of monitoring data is quantified, and forecasting based on history data is carried out. The experimental results on laboratory and field test data sets in M&F process show significant improvement in terms of regression with respect to the state-of-the-art GPz algorithm. VHGP can better capture the heteroscedastic variances of the noise, and the estimated confidence interval varies accordingly. For in-situ sensors, the accuracy improvement level varies, with an average increase of 66.1% on MSE. In regard to forecasting, the improvement is not as high as that of regression. VHGP is slightly superior to GPz in general, with a forecasting accuracy that increased by 21.24% on average for all sensors. However, the VHGP framework is more accurate for maximum noise position prediction, the forecasting mean of VHGP is more consistent with the real measurement values, and the method can yield more robust forecasting at the peaks for the field monitoring data. Finally, for the specific example of M&F for deformation of in-service HSR track, the uncertainty levels of all sensors are estimated with associated deformation profiles for the monitoring segment on the basis of in-situ measurement data, and three typical types of uncertainty, i.e., high uncertainty induced low data density, periodic uncertainty, and relatively stable uncertainty, are summarized during the M&F process of HSR track slab deformation. The purpose of uncertainty analysis is to quantify the uncertainty level of each sensor, to figure out the sensors with the maximum data volatility and the sensors with the most stable performance. During the modelling and forecastingfor the deformation of in-service HSR track slabs, the data of sensors with good stability shall be fully considered to obtain more accurate measurement results.

Future work will expand the monitoring system to deploy more FBG bending gauges along the longitudinal direction to cover the full length of the track slabs in the tunnel. The accuracy of multi-step ahead forecasting by VHGP method is not as high as that of one-step ahead forecasting. Further research will be pursued regarding how the sparse theory can be introduced into VHGP to improve its generalization capacity with the intention of achieving more accurate multi-step forecasting. The possible influence of covariance matrix selection on the result will also be studied. In addition, the HGP state-space spatiotemporal model will be investigated to deal with the multiple output problem.

## Figures and Tables

**Table 1:** MSE comparison between GPz and VHGP ($\times 10^{-5}$).

Method	Sensor No.		
	S2	S3	S4
GPz	13.939	12.794	15.425
VHGP	3.4559	5.1352	3.2719
